# Mesopancreas dissection level 3 for pancreatic head cancer in combined robotic/open pancreatoduodenectomy: a propensity score-matched study

**DOI:** 10.1007/s00464-024-11475-6

**Published:** 2024-12-29

**Authors:** Bor-Shiuan Shyr, Shin-E Wang, Shih-Chin Chen, Yi-Ming Shyr, Bor-Uei Shyr

**Affiliations:** 1 Division of General Surgery, Department of Surgery and Therapeutic and Research Center of Pancreatic Cancer, Taipei Veterans General Hospital, Taipei Veterans General Hospital, National Yang Ming University, 201 Section 2 Shipai Road, Taipei, 112 Taiwan, ROC; 2 National Yang Ming Chiao Tung University, Taipei, Taiwan, ROC

**Keywords:** Dissection, Level 3, Mesopancreas, Pancreatoduodenctomy, Robotic

## Abstract

**Background:**

Mesopancreas dissection (MPD) level 3 in combined robotic/open pancreatoduodenectomy (CR/OPD) is technique-demanding. This study aims to clarify the feasibility and justification of MPD level 3.

**Methods:**

Propensity score matching (PSM) analysis was conducted for 208 patients with pancreatic head cancer undergoing CR/OPD with or without MPD level 3. The comparison focused on surgical and oncological outcomes.

**Results:**

After PSM, each group comprised 86 patients. Surgical outcomes were comparable between these two groups, except longer operation time for MPD level 3 (+), median: 10.5 vs. 9.5 h, *p* = 0.002. MPD level 3 (+) group exhibited higher lymph node yield, median: 20 vs. 17, *p* < 0.001, and curative (R0) resection rate, 89.5% vs. 69.8%, *p* = 0.001, compared to MPD level 3 (−) group. Among the entire cohort, no significant survival difference was observed between the MPD Level 3 (+) and (−) groups. Survival outcome for R0 resection after CR/OPD was notably better than those for R2 resection, 5-year survival: 34.0% vs. 0, *p* = 0.038. However, within the curative (R0) resection cohort, no survival difference was observed between the MPD level 3 (+) and MPD level 3 (−) groups.

**Conclusion:**

MPD level 3 in CR/OPD is technically feasible without increasing the surgical risks but takes one hour extra operating time. Incorporation of MPD level 3 does not confer a survival advantage within the curative (R0) resection cohort. The primary focus should continue to be on achieving curative (R0) resection to maximize the survival benefits for pancreatic head cancer.

**Supplementary Information:**

The online version contains supplementary material available at 10.1007/s00464-024-11475-6.

Pancreatoduodenctomy stands as the single hope for achieving long-term survival in patients with pancreatic head cancer, and curative (R0) resection is the prime goal for this notorious malignancy. Unfortunately, only a limited percentage (15–25%) of patients with pancreatic head cancer are deemed eligible for curative resection at the initial diagnosis [1]. Recurrence is a prevalent occurrence, with rates potentially soaring up to 80% within the first year after curative-intent resection. This is often attributed to incomplete removal at the resection margins, implying that actual curative resection is not achieved in a significant proportion (20–86%) of pancreatic head cancer patients [2, 3]. The poor prognosis post-surgery is mainly linked to early lymph node involvement and the cancer's tendency to infiltrate retropancreatic tissues and spread along peripancreatic neural plexuses. Thus, Efforts to enhance R0 resection and improve survival outcomes necessitate extended radical resections, including extensive lymphadenectomy and dissection of the nerve plexus around the pancreas and superior mesenteric vessels [1, 4–8].

The concept of mesopancreas, first advocated by Gockel et al. [9], refers to retropancreatic tissue located between the superior mesenteric artery (SMA) and the pancreatic parenchyma, beginning from the posterior surface of the pancreatic head and extending behind the superior mesenteric vein (SMV) and SMA [2, 9, 10]. The mesopancreas without well-defined boundaries lacks fibrous sheaths and fascia [9–11]. It is composed of adipose areolar tissue, peripheral nerves, the pancreatic head nerve plexus, blood vessels, such as the inferior pancreatoduodenal arteries and veins, capillaries, lymphatics and lymph nodes [1, 2, 5–10, 12, 13]. The term “mesopancreas dissection (MPD)” proposed by Inoue et al. [5] is used to describe the extent of lymph node dissection. MPD level 3 involving en bloc mesopancreas resection and extended dissection along the SMA has been described in detail by our previous study and literature [1, 5, 7]. Pancreatic head cancer tends to have lymph node involvement around the pancreas and perineural invasion around SMA and in the mesopancreas [1, 5]. Therefore, the rationale for extended radical resection is to increase local radicality, including en bloc mesopancreas and nerve plexus around the SMA, particularly for those with borderline resectable pancreatic head cancer with direct or indirect invasion to major vessels [5–8, 13–15]. However, such extended resection might increase the surgical risks, especially intractable diarrhea, chyle leakage, and delayed gastric emptying (DGE) [1, 7, 8, 14]. There are some studies investigating the MPD level 3, but there is ongoing controversy regarding the safety and contribution to the long-term survival of patients with pancreatic cancer [4, 7, 16–21]. Therefore, the justification of extended radical resection with MPD level 3 and its beneficial effects remain controversial.

The introduction of the da Vinci Robotic Surgical System (Intuitive Surgical®, Sunnyvale, CA, USA) has opened avenues for robotic pancreatoduodenctomy (CR/OPD). Despite concerns about the high cost and technical challenges, our previous study demonstrated the feasibility of MPD level 3 in CR/OPD without compromising the safety [1]. This approach could serve as a safe alternative to open pancreatoduodenctomy for extensive radical dissection.

To further investigate the oncological justification and technical feasibility of MPD level 3 in CR/OPD, we conducted a propensity score matching (PSM) study comparing groups of CR/OPD with and without MPD level 3. This study aims to contribute valuable insights into the safety and potential benefits of extended radical resection in patients with pancreatic head cancer undergoing robotic surgery.

## Methods

Data from patients with pancreatic head adenocarcinoma who underwent CR/OPD between 2012 and 2023 were identified for this PSM comparative study from a prospectively collected computer database. The study exclusively focused on pancreatic head adenocarcinoma cases only. Approval for this study was obtained from the Institutional Review Board (IRB) (IRB-TPEVGH No.: 2023-11-013CC) at Taipei Veterans General Hospital, and the research adhered to IRB regulations and guidelines. Informed consent requirements were waived due to the anonymized and retrospective nature of the study.

The da Vinci Si or Xi Surgical System (Intuitive Surgical, Inc., Sunnyvale, CA, USA) was utilized for CR/OPD. Patient selection for CR/OPD was based on individual preferences following comprehensive counseling about the benefits and drawbacks of this innovative surgical approach. Patients bore the cost of robotic surgery, and the CR/OPD scheduling was contingent upon the availability of the robotic system. Exclusion criteria for CR/OPD encompassed long vascular encasement (> 2 cm) by cancer and severe intraabdominal adhesions. All perioperative data were prospectively collected and stored in a computer database for this retrospective PSM study, covering demographics, surgical outcomes, oncological and pathological parameters, and other relevant surgical details.

### Study endpoints

The objectives of this study were to evaluate the feasibility and rationale behind implementing MPD level 3 for pancreatic head cancer in CR/OPD. The primary study endpoint aimed to compare the oncological and survival outcomes for pancreatic head cancer in the study group of MPD level 3 (+) with the control group of MPD level 3 (−) in CR/OPD. The secondary study endpoint involved assessing the surgical risks associated with CR/OPD, both with and without the incorporation of MPD level 3.

### Propensity score matching (PSM) strategy

The PSM was implemented to mitigate selection bias and ensure equitable comparisons while assessing the causal treatment effects within the study cohort of CR/OPD with MPD level 3 and the control cohort of CR/OPD without MPD level 3. This methodology involved the generation of individual propensity scores through logistic regression modeling, utilizing six covariates commonly employed for predicting prognosis in pancreatic adenocarcinoma: (1) tumor size, (2) lymph node involvement, (3) lymphovascular invasion, (4) perineural invasion, (5) differentiation of tumor cells, and (6) tumor stage. Subsequently, patients from the CR/OPD with MPD level 3 (+) and MPD level 3 (−) were paired in a 1:1 ratio. Matching commenced with cases having the highest propensity scores, and a specific caliper width of 0.2 standard deviations of the logit of the estimated propensity score was applied during the PSM process.

### Mesopancreas dissection

**Fig. 1:**
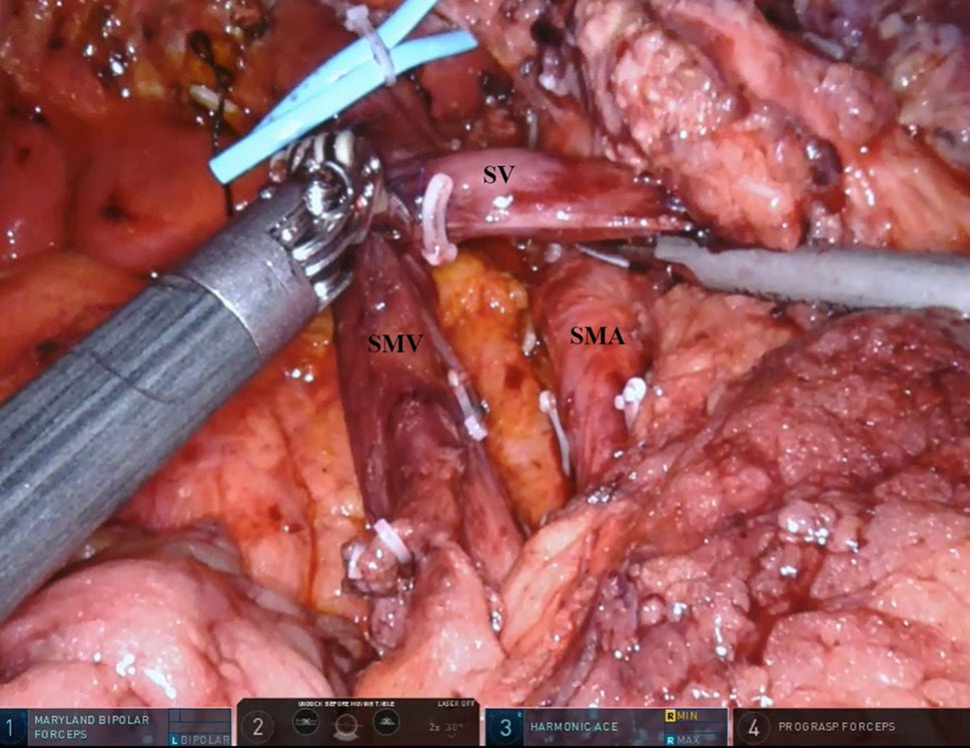
Mesopancreas level 3 dissection in combined robotic/open pancreatoduodenectomy for a pancreatic head adenocarcinoma. En bloc mesopancreas resection with periadventitial dissection including nerve plexus along the right side of SMA. SMA, superior mesenteric artery; SMV, superior mesenteric vein; SV, splenic vein

According to the extent of dissection around and along the SMA and SMV, MPD was categorized into three levels: Level 1: mesopancreas division along the right side of SMV; Level 2: mesopancreas division along the right side (from 5 to 11 o’clock) of the SMA, leading to en bloc resection of the corresponding lymph nodes and mesojejunum. This level does not include the nerve plexus around the SMA; Level 3: En bloc mesopancreas resection with periadventitial dissection, including the nerve plexus along the right side of the SMA (Fig. 1). The details of robotic mesopancreas dissection level are shown by the attached video clip, which also can be displayed by scanning the attached QR code. This comprehensive level involves a more extensive resection [5–7]. Before 2018, only level 1 and 2 mesopancreas dissections were performed at our institute. However, since 2018, after accumulating experience in CR/OPD, MPD level 3 has been routinely performed for pancreatic head adenocarcinoma whenever possible. For detailed information on MPD, refer to our previous study [1].

### Surgical technique

CR/OPDs with and without MPD level 3 were consistently performed by the same pancreatic surgical team, led by Dr. Shyr YM and Dr. Wang SE. The details of pancreatic reconstruction using the modified Blumgart technique have been described in our previous study [22, 23]. The Si or Xi da Vinci Surgical System® (Intuitive Surgical, Inc., Sunnyvale, CA, USA) was utilized to facilitate CR/OPD. The Harmonic® scalpel, an energy device, was employed for the division of small vessels, while Hem-o-lok® systems (Teleflex Inc., Chelmsford, MA, USA) were selectively used for the management of larger vessels. The final anastomosis, a hand-sewn gastrojejunostomy, was performed extracorporeally through a 3–6 cm umbilical wound, which was also used for the extraction of the surgical specimen. Therefore, based on the Brescia consensus [24], our technique is a type I combined robotic/open procedure, in which only the final anastomosis, gastrojejunostomy, was by open approach. Neoadjuvant chemotherapy was not applied for the patients in this study, but adjuvant chemotherapy was routinely administered for pancreatic cancer after CR/OPD whenever possible, regardless of the cancer stage. This comprehensive approach aims to enhance the overall management and outcomes of patients undergoing robotic pancreatoduodenctomy.

### Definitions of surgical complications

Delayed gastric emptying (DGE) was defined according to the grading system established by the International Study Group of Pancreatic Surgery (ISGPS), specifically referring to Grade B or C complications (ISGPS grade) [25]. Postoperative pancreatic fistula (POPF) was identified as clinically relevant Grade B or C, in adherence to the 2016 grading system revised by the International Study Group for Pancreatic Fistula (ISGPF) [26]. Postpancreatectomy hemorrhage (PPH) and chyle leak were categorized based on the criteria outlined by the ISGPS [27, 28]. Resection margins refer to pancreatic cutend, biliary cutend, vascular groove, uncinate process, pancreatic anterior surface and pancreatic posterior surface. Resection radicality was stratified into three distinct categories, determined by the resection margin status: R0: A resection without gross and microscopic evidence of cancer at the resection margin, with a margin > 1 mm; R1: A resection characterized by grossly negative but microscopically positive cancer at the resection margin, with a margin ≤ 1 mm; and R2: A resection demonstrating grossly positive cancer at the resection margin. Surgical mortality was defined as any death occurring within 90 days after surgery, encompassing both the period of admission for the initial operation and any subsequent hospital readmission. This comprehensive set of definitions ensures a standardized and consistent assessment of surgical outcomes and complications in the context of pancreatic surgery.

### Statistical analysis

Statistical analyses were conducted using version 26.0 of Statistical Product and Service Solutions (SPSS Inc., IBM, Armonk, NY, USA). Categorical variables were presented as numbers and percentages, and comparisons were made using either Pearson's χ2 test or Fisher's exact test in contingency tables. Continuous data were described using median, range, mean ± standard deviation (SD). The means of two groups were compared using the two-tailed Student’s t-test. In instances where continuous variables did not follow a normal distribution, the Wilcoxon rank-sum test was applied. Survival rates were estimated using the Kaplan–Meier method, and differences between the survival curves were assessed using the log-rank test. A *p*-value less than 0.05 was considered statistically significant, indicating meaningful differences between the groups under investigation.

## Results

A total of 208 patients with pancreatic head cancer underwent CR/OPD, with 88 undergoing MPD level 3 and 120 without MPD level 3. Following PSM, each group comprised 86 patients. The demographic characteristics of the patients are summarized in Table 1. No significant differences were observed in terms of sex, age, body mass index, American Society of Anesthesiologists physical status, jaundice, body weight loss, pancreas parenchyma, pancreatic duct, and tumor size between the two groups. However, there were more patients with diabetes mellitus in the MPD level 3 (+) group, both before and after PSM.

**Table 1:** Demographics of patients with pancreatic head cancer undergoing robotic pancreaticoduodenectomy

	Before propensity score-matching		*p* value	After propensity score-matching		*p* value
	With MPD level 3	without MPD level 3		With MPD level 3	without MPD level 3	
Patients, n	88	120		86	86	
Sex			0.552			0.879
Female	44 (50.0%)	55 (45.8%)		43 (50.0%)	44 (81.2%)	
Male	44 (50.0%)	65 (54.2%)		43 (50.0%)	42 (48.8%)	
Age, year old			0.439			0.390
Median (range)	70 (41–89)	69 (27–97)		70 (41–89)	71 (27–97)	
Mean ± SD	68 ± 10	69 ± 12		68 ± 10	69 ± 12	
BMI			0.963			0.779
Median (range)	24 (17–36)	24 (16–35)		24 (17–36)	23 (16–35)	
Mean ± SD	24 ± 4	24 ± 4		24 ± 4	24 ± 4	
ASAPS classification ≥ 3	38 (43.2%)	50 (41.7%)	0.827	38 (44.2%)	43 (50.0%)	0.445
Diabetes mellitus	39 (44.3%)	36 (30.0%)	0.034	38 (44.2%)	23 (26.7%)	0.017
Jaundice	56 (63.6%)	89 (74.2%)	0.102	56 (65.1%)	66 (76.7%)	0.093
Body weight loss	35 (39.8%)	37 (30.8%)	0.181	34 (39.5%)	24 (27.9%)	0.107
Pancreas parenchyma			0.991			0.872
Soft	30 (34.1%)	41 (34.2%)		30 (34.9%)	29 (33.7%)	
Hard	58 (65.9%)	79 (65.8%)		56 (69.1%)	57 (66.3%)	
Pancreatic duct			0.418			0.737
Non-dilated ≤ 3 mm	24 (27.3%)	39 (32.5%)		24 (27.9%)	26 (30.2%)	
Dilated > 3 mm	64 (72.7%)	81 (67.5%)		62 (72.1%)	60 (69.8%)	
Tumor size, cm			0.942			0.619
Median (range)	3.0 (1.7–6.5)	3.0 (1.3–11.0)		3.0 (1.7–6.0)	3.0 (1.3–11.0)	
Mean ± SD	3.2 ± 1.0	3.2 ± 1.1		3.1 ± 0.9	3.0 ± 1.1	

MPD, mesopancreas dissection; SD, standard deviation; BMI, body mass index; ASAPS, American Society of Anesthesiologists physical status

The surgical outcomes are detailed in Table 2. The operation time for CR/OPD with MPD level 3 (+) was prolonged by 1 h compared to MPD level 3 (−), with a median duration of 10.5 vs. 9.5 h, *p* = 0.002 after PSM. The vascular resection rate was higher in the MPD level 3 (+) group, at 27.9%, compared to 15.1% in the MPD level 3 (−) group, *p* = 0.041. However, other perioperative surgical outcomes, including intraoperative blood loss, open conversion rate, surgical mortality and morbidity, postoperative pancreatic fistula (POPF), delayed gastric emptying (DGE), and postpancreatectomy hemorrhage (PPH), demonstrated comparability between the MPD level 3 (+) and MPD level 3 (−) groups.

**Table 2:** Surgical outcomes for patients with pancreatic head cancer undergoing robotic pancreaticoduodenectomy

	Before propensity score-matching		*p* value	After propensity score-matching		*p* value
	With MPD level 3	Without MPD level 3		With MPD level 3	Without MPD level 3	
Patients, *n* (%)	88	120		86	86	
Operation time, hour			< 0.001			0.002
Median (range)	10.5 (6.0–16.0)	9.3 (8–15.3)		10.5 (6.0–16.0)	9.5 (5.2–14.0)	
Mean ± SD	10.5 ± 2.0	9.4 ± 2.3		10.5 ± 2.0	9.5 ± 2.2	
Blood loss, c.c			0.156			0.379
Median (range)	200 (29–1000)	208 (0–2700)		200 (29–1000)	200 (0–1600)	
Mean ± SD	272 ± 215	330 ± 340		270 ± 217	303 ± 265	
Conversion to open, n (%)	15 (17.0%)	21 (17.5%)	0.932	13 (15.1%)	14 (16.3%)	0.834
Surgical mortality	2 (2.3%)	0	0.097	2 (2.3%)	0	0.497
Surgical morbidity	53 (60.2%)	60 (50.0%)	0.143	52 (60.5%)	44 (51.2%)	0.219
Complication classification			0.107			0.191
Clavien–Dindo 0	35 (39.8%)	60 (50.0%)		34 (39.5%)	42 (48.8%)	
Clavien–Dindo I	35 (39.8%)	32 (26.7%)		35 (40.7%)	24 (27.9%)	
Clavien–Dindo II	7 (8.0%)	12 (10.0%)		7 (8.1%)	10 (11.6%)	
Clavien–Dindo III	9 (10.2%)	134 (10.8%)		8 (9.3%)	8 (9.3%)	
Clavien–Dindo IV	0	3 (2.5%)		0	2 (2.3%)	
Clavien–Dindo V (death)	2 (2.3%)	0		2 (2.3%)	0	
Severity of complication			0.462			0.431
Minor (Clavien–Dindo I-II)	11 (20.8%)	16 (26.7%)		42 (48.8%)	34 (39.5%)	
Major (Clavien–Dindo ≥ III)				10 (11.6%)	10 (11.6%)	
POPF (grade B and C)	7 (8.0%)	5 (4.2%)	0.247	7 (8.1%)	4 (4.7%)	0.350
Pancreas parenchyma						
Soft	6 (20.0%)	3 (7.3%)	0.113	6 (20.0%)	2 (6.9%)	0.254
Hard	1 (1.7%)	2 (2.5%)	0.750	1 (1.8%)	2 (3.5%)	1.000
Pancreatic duct						
Non-dilated ≤ 3 mm	3 (12.5%)	3 (7.7%)	0.528	3 (12.5%)	2 (7.7%)	0.571
Dilated > 3 mm	4 (6.3%)	2 (2.5%)	0.256	4 (6.5%)	2 (3.3%)	0.426
DGE	3 (3.4%)	7 (5.8%)	0.419	3 (3.5%)	5 (5.8%)	0.469
PPH	6 (6.8%)	5 (4.2%)	0.399	6 (7.0%)	4 (4.7%)	0.515
Chyle leakage	28 (31.8%)	32 (26.7%)	0.418	28 (32.6%)	24 (27.9%)	0.205
Bile leakage	1 (1.1%)	2 (1.7%)	0.751	1 (1.2%)	1 (1.2%)	1.000
Wound infection	4 (4.5%)	7 (5.8%)	0.682	4 (4.7%)	5 (5.8%)	0.732

MPD, mesopancreas dissection; SD, standard deviation; POPF, postoperative pancreatic fistula; DGE, delayed gastric emptying; PPH, postpancreatectomy hemorrhage

The lymph node yield in the MPD Level 3 (+) group was significantly higher than that in the MPD Level 3 (−) group, with a median of 21 vs. 17, *p* < 0.001 before PSM and 20 vs. 17, *p* < 0.001 after PSM (Table 3). Additionally, the curative (R0) resection rate was notably elevated in the MPD Level 3 (+) group compared to the MPD Level 3 (−) group, at 89.8% vs. 70.8%, *p* = 0.001 before PSM and 89.5% vs. 69.8%, *p* = 0.001 after PSM. Importantly, after PSM, there were no significant differences in pathological outcomes between the two groups, including lymph node involvement, lymphovascular invasion, perineural invasion, differentiation of tumor cells, and stage.

**Table 3:** Oncological and pathological outcomes for patients with pancreatic head cancer undergoing robotic pancreaticoduodenectomy

	Before propensity score-matching		*p* value	After propensity score-matching		*p* value
	With MPD level 3	Without MPD level 3		With MPD level 3	Without MPD level 3	
Patients, *n* (%)	88	120		86	86	
Lymph node yield, n			< 0.001			< 0.001
Median (range)	21 (8–37)	17 (3–43)		20 (8–37)	17 (3–30)	
Mean ± SD	21 ± 6	17 ± 7		21 ± 6	16 ± 6	
Radicality of resection						0.001
R0	79 (89.8%)	85 (70.8%)	0.001	77 (89.5%)	60 (69.8%)	
R1	8 (9.1%)	16 (13.3%)		8 (9.3%)	11 (12.8%)	
R2	1 (1.1%)	19 (15.8%)		1 (1.2%)	15 (17.4%)	
Vascular resection	25 (28.4%)	20 (16.7%)	0.042	24 (27.9%)	13 (15.1%)	0.041
Lymph node involvement	61 (69.3%)	84 (70.0%)	0.916	55 (64.0%)	62 (72.1%)	0.250
Lymph node involvement	62 (70.5%)	105 (87.5%)	0.002	71 (82.6%)	61 (70.9%)	0.071
Perineural invasion	80 (90.9%)	110 (91.7%)	0.848	78 (90.7%)	78 (90.7%)	1.000
Differentiation of tumor cell			0.362			0.114
Well	9 (10.2%)	8 (6.7%)		9 (10.5%)	8 (9.3%)	
Moderate	59 (67.0%)	91 (75.8%)		61 (70.9%)	71 (82.6%)	
Poor	20 (22.7%)	21 (17.5%)		16 (18.6%)	7 (8.1%)	
Stage			0.642			0.826
I + II	65 (73.9%)	92 (76.7%)		63 (74.1%)	61 (72.6%)	
III + IV	23 (26.1%)	28 (23.3%)		22 (25.9%)	23 (27.4%)	

MPD, mesopancreas dissection; SD, standard deviation; R0, curative resection with a margin > 1 mm, R1, microscopic residual cancer ≤ 1 mm; R2, gross residual cancer

**Fig. 2:**
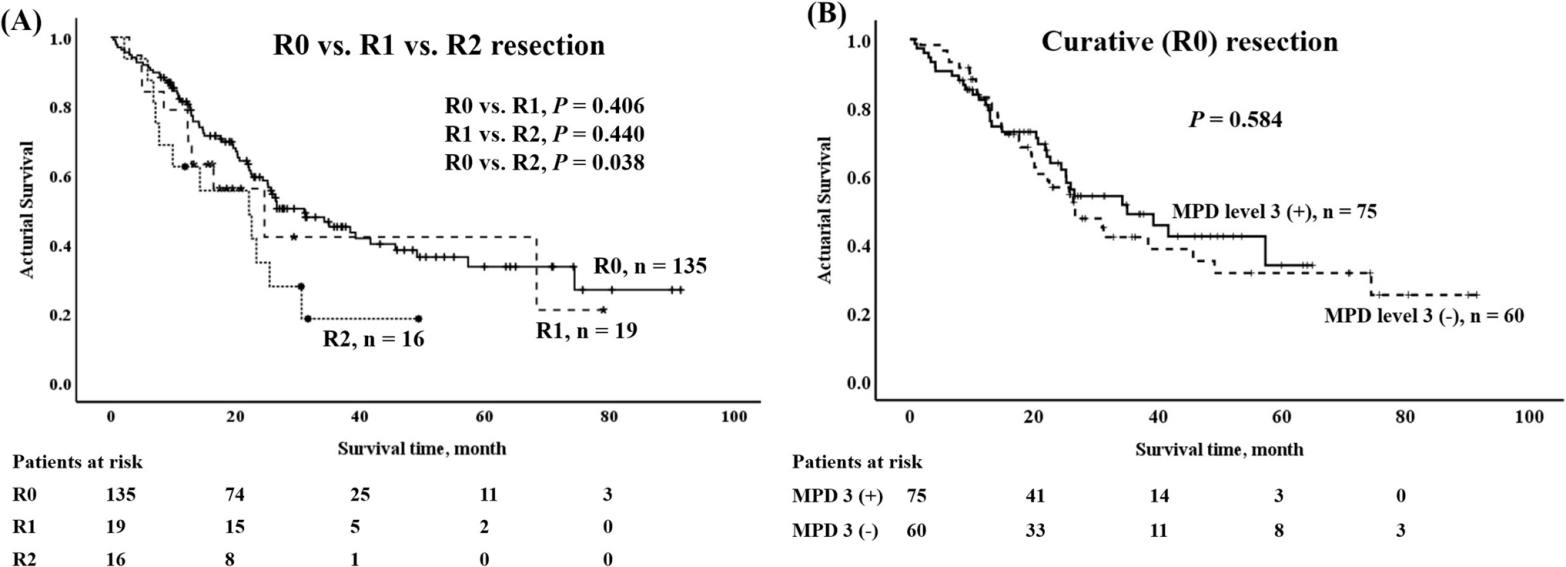
**A** Survival curves for pancreas head cancer after combined robotic/open pancreatoduodenectomy R0 vs. R2, *p* = 0.038. **B** Survival curves for pancreas head cancer after combined robotic/open pancreatoduodenectomy with mesopancreas dissection (MPD) level 3 vs. without MPD level 3, *p* = 0.584

Survival outcomes for pancreatic head cancer following CR/OPD after PSM are presented in Table 3. The 5-year survival rate for all patients was 32.4%, with a median survival of 20.3 months and a mean of 25.1 ± 19.3 months. Among the entire cohort of pancreatic head cancer patients who underwent CR/OPD, no significant survival difference was observed between the MPD Level 3 (+) and MPD Level 3 (−) groups, with a *p* value of 0.257. Comparatively, the survival outcome for R0 (curative) resection in pancreatic head cancer after CR/OPD was markedly superior to that of R2 resection, exhibiting a 5-year survival of 34.0% vs. 0, with a *p* value of 0.038 (Fig. 2A). Nevertheless, within the R0 (curative) resection subgroup, no discernible survival difference was noted between the MPD Level 3 (+) and MPD Level 3 (−) groups, with a* p* value of 0.258 (Fig. 2B). These findings suggest that while curative resection significantly improves survival outcomes compared to palliative resection, the addition of MPD Level 3 did not yield a survival advantage within the curative (R0) resection cohort.

## Discussion

Curative resection (R0) represents the primary avenue for curing pancreatic cancer patients. However, numerous curative-intent resections for pancreatic head cancer are ultimately classified as R1 resections upon histopathological examination. This disappointing outcome underscores the necessity for pancreatic surgeons to refocus their attention on the retropancreatic resection margin, a frequent site leading to R1 resection in pancreatic head cancer cases. To enhance the R0 resection rate, some authors advocate for extended radical resection to achieve negative margins toward SMV and SMA. These vessels are often identified as crucial challenging areas during pancreatoduodenctomy for pancreatic head cancer [5–7]. The intriguing concept of the "mesopancreas" has been introduced to characterize the retropancreatic tissue situated between the SMA and the pancreatic parenchyma [1, 2, 5–10, 12, 13]. Inoue et al. [5] proposed the term "MPD" to delineate the extent of lymph node dissection, with MPD level 3 involving en bloc mesopancreas resection and extended dissection along the SMA [1, 5, 7].

Pancreatoduodenctomy is a technically demanding and time-consuming surgical procedure. However, achieving MPD level 3 during pancreatoduodenctomy poses inherent technical challenges, even with the traditional open approach, and particularly with the robotic approach. Our study revealed that performing CR/OPD with MPD level 3 (+) resulted in a longer operation time compared to CR/OPD with MPD level 3 (−). However, MPD level 3 in CR/OPD did not elevate surgical risks, including blood loss, open conversion, surgical mortality, morbidity, POPF, DGE, PPH, chyle leakage, bile leakage, and wound infection. Importantly, existing evidence indicates that pancreatoduodenctomy with extended lymphadenectomy does not necessarily increase the risks of perioperative morbidity and mortality in specialized centers when compared to standard resections [5–7]. In other words, our findings support the feasibility of MPD level 3 in CR/OPD without compromising safety.

The SMA margin typically emerges as the predominantly positive margin following standard resection for pancreatic head cancer, with numerous prospective and retrospective studies consistently indicating enhanced survival outcomes in R0 resections featuring negative margins [29]. While survival predictions for pancreatic cancer often hinge on total harvested lymph node counts [30], the underlying mechanism remains elusive. This study underscores the significance of MPD level 3 (+), revealing a notable increase in R0 resection rate with margins > 1 mm, as compared to MPD level 3 (−) cases (89.5% vs. 69.8%, *p* = 0.001). Additionally, there is a considerable rise in lymph node yield (20 vs. 17, *p* < 0.001) and vascular resection rate (27.9% vs. 15.1%, *p* = 0.041) in MPD level 3 (+). The heightened vascular resection rate potentially contributes to the increased R0 resection rate observed in MPD level 3 (+). Given that the positive impact of MPD level 3 on R0 resection rate and lymph node yield, an assertive approach to MPD level 3 dissection, inclusive of vascular resection, is justified for achieving oncological radicality in CR/OPD. This study marks a pioneering effort in utilizing PSM to elucidate the feasibility and oncological justification of MPD level 3 dissection in the context of CR/OPD.

The optimal extent of radical dissection in pancreatic cancer remains a subject of debate. Recently, there has been a growing belief that R1 resections often result from incomplete mesopancreas resection in patients with pancreatic head cancer [2, 13]. Accordingly, some authors have proposed total mesopancreas excision with extensive dissection to enhance the R0 resection rate and improve the prognosis of pancreatic cancer [2, 8, 13]. However, despite these propositions, there is currently no widespread consensus on whether extensive lymph node dissection provides a survival benefit for pancreatic head cancer, as indicated by previous randomized controlled trials [4, 7, 17, 19–21]. Furthermore, meta-analyses of both randomized controlled trials (RCTs) and non-RCTs evaluating extended lymphadenectomy in pancreatoduodenctomy have failed to demonstrate an improvement in overall survival compared to standard lymphadenectomy [10]. Consistent with expectations, this study reveals superior survival outcomes in the curative (R0) resection group compared to the R2 group, whereas a 5-year survival is also feasible with R1 resection as shown in this study. Probably tumor biology in general and occult micrometastatic disease are much more relevant than local disease control. However, among patients with curative (R0) resection after PSM, there is no discernible survival difference between MPD level 3 (+) and (−) groups. These findings suggest that while curative resection significantly enhances survival outcomes compared to palliative resection, the inclusion of MPD Level 3 does not confer a survival advantage within the curative (R0) resection cohort. Consequently, achieving curative (R0) resection should remain the primary objective to maximize the survival benefits for pancreatic head cancer, irrespective of the level of mesopancreas dissection in CR/OPD.

## Limitations

This study has certain limitations. In contrast to randomized controlled trials, the PSM analysis employed in this study considers only six covariates that are commonly used to predict prognosis in pancreatic cancer. Unobserved confounders that were not accounted for in this analysis may still be present, potentially leading to biased results. Moreover, other confounding factors not addressed in the PSM analysis could also influence our findings. Consequently, large-scale prospective randomized controlled trials with extended follow-up periods are imperative to validate the impact of MPD level 3 on both surgical and oncological outcomes for pancreatic head cancer in CR/OPD.

## Conclusion

MPD Level 3 in CR/OPD is technically feasible without increasing surgical risks. The data of this study support that MPD Level 3 enhances the rate of achieving R0 resection with a free margin > 1 mm and increases lymph node yield. Considering these benefits, an aggressive approach to MPD Level 3, including vascular resection, can be justified for achieving oncological radicality in CR/OPD. While curative resection significantly enhances survival outcomes compared to palliative resection, the study suggests that the addition of MPD Level 3 does not provide a survival advantage within the curative (R0) resection cohort. Consequently, MPD Level 3 is not routinely recommended for pancreatic cancer in CR/OPD, and achieving curative (R0) resection should remain the prime goal to maximize the survival benefits for pancreatic head cancer, irrespective of the level of mesopancreas dissection in CR/OPD.

## Supplementary Information

Below is the link to the electronic supplementary material.

Supplementary file1 (MP4 183544 KB)

Supplementary file2 (PNG 6 KB)
