# Supplementary figures and images for: Mesopancreas dissection level 3 for pancreatic head cancer in combined robotic/open pancreatoduodenectomy: a propensity score-matched study
Source: Surg Endosc. 2024 Dec 29;39(2):1191–9. doi: 10.1007/s00464-024-11475-6 (PMC11794409; doi:10.1007/s00464-024-11475-6)

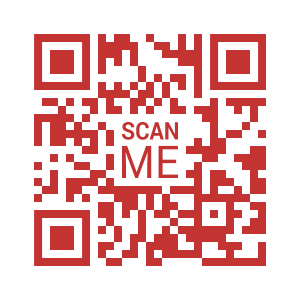

Supplement: Supplementary file 2 — Supplementary file2 (PNG 6 KB) [file 464_2024_11475_MOESM2_ESM.png]
